# Placental Endocannabinoid System: Focus on Preeclampsia and Cannabis Use

**DOI:** 10.1161/HYPERTENSIONAHA.125.24934

**Published:** 2025-04-17

**Authors:** Madhavi S. Harhangi, Sinno H.P. Simons, Hilmar H. Bijma, Anna Nguyen, Tuong-Vi Nguyen, Tu’uhevaha Kaitu’u-Lino, Irwin K.M. Reiss, A.H. Jan Danser, Michelle Broekhuizen

**Affiliations:** Division of Neonatology, Department of Neonatal and Pediatric Intensive Care (M.S.H., S.H.P.S., I.K.M.R., M.B.), Erasmus MC University Medical Center, Rotterdam, The Netherlands.; Division of Pharmacology and Vascular Medicine, Department of Internal Medicine (M.S.H., A.H.J.D., M.B.), Erasmus MC University Medical Center, Rotterdam, The Netherlands.; Division of Obstetrics and Fetal Medicine (M.S.H., H.H.B.), Erasmus MC University Medical Center, Rotterdam, The Netherlands.; Mercy Hospital for Women, Department of Obstetrics, Gynaecology and Newborn Health, The University of Melbourne, Australia (A.N., T.-V.N., T.K.-L.).

**Keywords:** anandamide, cannabis, endocannabinoids, placenta, pre-eclampsia

## Abstract

**BACKGROUND:**

The endocannabinoid system (ECS) plays an important role in the early stages of pregnancy, while cannabis use during pregnancy associates with a greater risk of preeclampsia. This study quantified the placental ECS component mRNA levels in gestational age-matched healthy pregnant women, women with preeclampsia, and women who used cannabis throughout their pregnancy. Next, it compared the effects of the endogenous ECS agonists anandamide and 2-arachidonoylglycerol with those of the cannabinoid receptor type 1 and 2 agonists HU-210 and HU-308 in chorionic plate arteries.

**METHODS:**

Placental mRNA levels were quantified by quantitative polymerase chain reaction. Vascular reactivity was studied with and without selective cannabinoid receptor type 1 and 2 antagonists.

**RESULTS:**

mRNA levels of 1,2-diacylglycerol lipase α, responsible for 2-arachidonoylglycerol generation, were lowered in preeclampsia, while mRNA levels of the anandamide-synthesizing enzyme *N*-acyl phosphatidylethanolamine-specific phospholipase D were upregulated in cannabis users. Anandamide-induced relaxation in healthy pregnancy was mediated via cannabinoid receptors type 1 and 2, while 2-arachidonoylglycerol induced relaxation via cannabinoid receptor type 1. In preeclampsia, the effects of anandamide and 2-arachidonoylglycerol were unaltered but no longer involved cannabinoid receptors, while in cannabis users their effects were absent. HU-210 and HU-308 relaxed healthy, but not preeclamptic vessels. The NO donor sodium nitroprusside similarly relaxed healthy and preeclamptic vessels, while its effects in cannabis users were greatly reduced.

**CONCLUSIONS:**

The ECS is disturbed in preeclampsia, and endogenous ECS agonists lose their capacity to dilate in cannabis users, while such use also diminishes NO signaling. These data provide mechanistic evidence against cannabis use during pregnancy.

**NOVELTY AND RELEVANCE**

**What Is New?**

The placental mRNA levels of endocannabinoid system components are altered in preeclampsia and pregnant women using cannabis, implying that also the generation of the endocannabinoid agonists anandamide and 2-arachidonoylglycerol might be altered in the placenta of these women.

In healthy chorionic plate arteries, anandamide and 2-arachidonoylglycerol induced relaxation via cannabinoid receptor types 1 and 2, while in preeclampsia, their relaxant effects were intact but no longer involved cannabinoid receptors. In pregnant women using cannabis, their relaxant effects were absent.

Although the nitric oxide donor sodium nitroprusside relaxed healthy and preeclamptic vessels identically, its effect in cannabis users was greatly diminished.

**What Is Relevant?**

These data warn against the use of cannabis and cannabis-containing oils in pregnancy.

They also explain why such use is associated with preeclampsia.

**Clinical/Pathophysiological Implications?**

Pregnant women should actively be made aware of the potential deleterious effects of cannabis use, particularly because such use often occurs without informing their gynecologist.

The endocannabinoid system (ECS) is expressed throughout the human body.^1,2^ Its effects are mediated via 2 G-protein coupled receptors, namely CB1 (cannabinoid receptor type 1) and CB2 (cannabinoid receptor type 2; Figure 1).^3^ Their endogenous ligands (the so-called endocannabinoids or eCBs) are anandamide and 2-arachidonoylglycerol (2-AG). However, anandamide also binds to the transient potential receptor of the TRPV1 (vanilloid type 1 channel) and PPARγ (peroxisome proliferator-activated receptor gamma),^4,5^ while 2-AG may additionally activate the widely expressed orphan receptor GPR55 (G-protein coupled receptor 55).^6,7^ The physiological relevance of this alternative agonism is still unclear.

**Figure 1.**
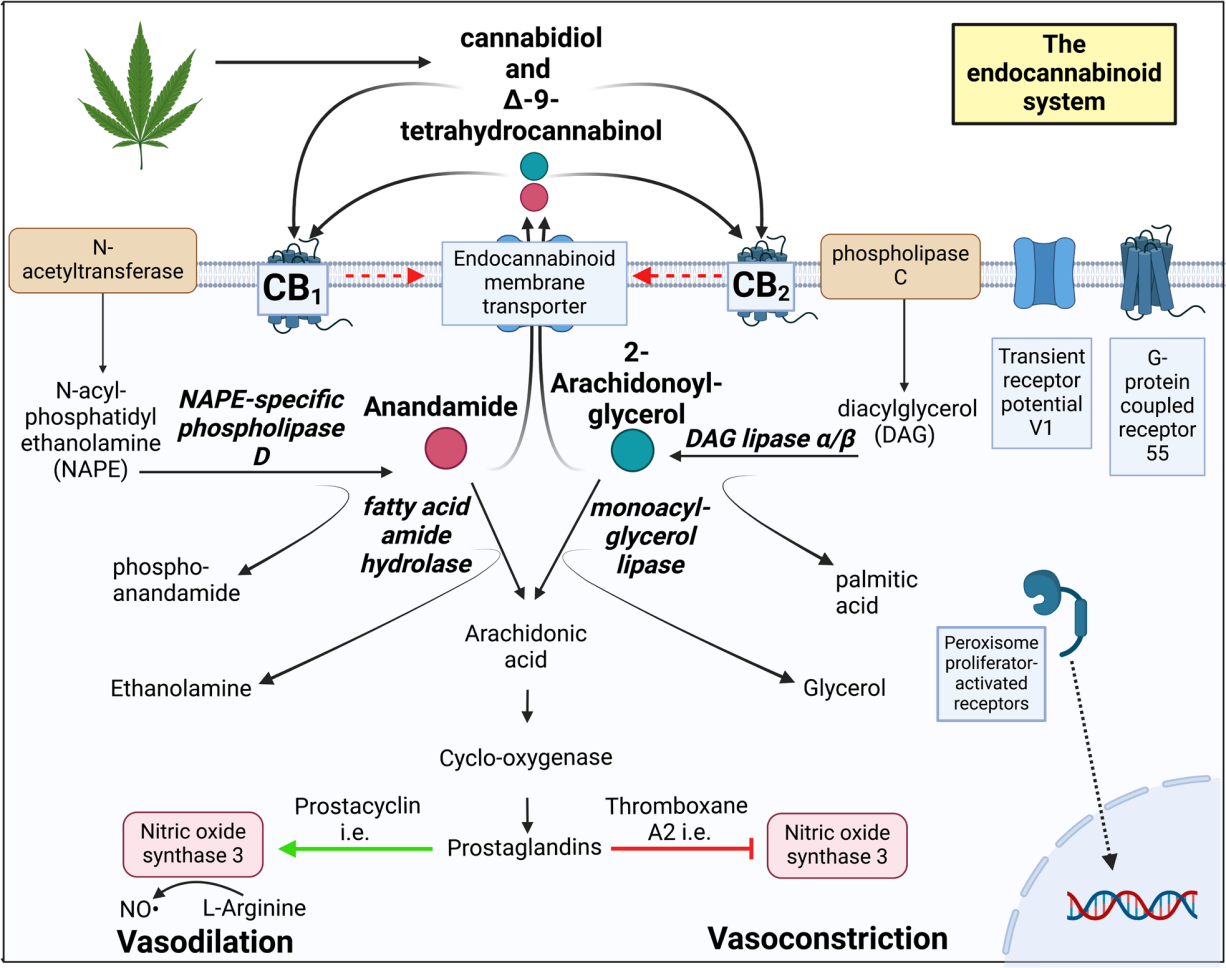
**Overview of the production and degradation of endocannabinoids and the potential influence of exogenous cannabis components on this system, as well as their interaction with cannabinoid receptors 1 and 2 (CB1 and CB2).** Created in BioRender. Harhangi, M.

*N*-acyl phosphatidylethanolamine-specific phospholipase D (NAPE-PLD) and 1,2-diacylglycerol lipase α/β (DAGLα/β) generate eCBs from phospholipids (Figure 1), while fatty acid amide hydrolase (FAAH) and monoacylglycerol lipase (MAGL) metabolize eCBs to arachidonic acid.^3^ COX (cyclooxygenase) subsequently converts arachidonic acid to prostaglandins, including the vasodilator prostacyclin and the vasoconstrictor thromboxane A2, thus providing a link between eCBs and vasoreactivity. Yet, anandamide also dilates human mesenteric,^8^ human pulmonary^9^ arteries, and a wide range of nonhuman vessels directly,^10–12^ and this is also true for 2-AG.^13,14^ Both the CB1^12^ and CB2^15^ receptors might be involved, and the described second messenger pathways include mitogen-activated protein kinase, nitric oxide (NO),^8^ calcium,^16^ and the big conductance calcium-activated potassium channel.^17^

In the placenta, the ECS contributes to embryo implantation, placentation, and trophoblast differentiation,^18,19^ while eCB production varies with gestational age (GA).^20,21^ Cannabis components like cannabidiol and Δ^9^-tetrahydrocannabinol affect the ECS through their binding to CB receptors.^22^ Cannabis use, as well as the use of cannabidiol-containing oils, is on the rise during pregnancy.^23^ This is mainly due to the increased legalization of cannabis use worldwide.^23^ Moreover, it is believed to alleviate morning sickness.^24^ However, such use is also associated with maternal and fetal complications,^25,26^ like fetal growth restriction (FGR), gestational hypertension, and preeclampsia.^27,28^ The latter relationship concurs with growing evidence that the ECS plays a role in the pathophysiology of PE.^29^ For instance, increased CB1 receptor mRNA levels have been reported in the placentas of women with PE.^30,31^

In the present study, we hypothesized that the placental ECS is altered by both PE and cannabis use and that this would diminish the dilator response to its endogenous ligands, anandamide and 2-AG. To address this hypothesis, we first evaluated the placental ECS in healthy pregnant women, women with early onset preeclampsia (eoPE) with or without FGR, and women who used cannabis during their pregnancy. Given the eCB changes throughout pregnancy, we took great care to obtain GA-matched healthy controls. Second, since the effects of anandamide and 2-AG on the contractility of placental vessels are currently unknown, we evaluated these effects in feto-placental arteries obtained from healthy pregnant women, women with eoPE, and women who used cannabis during their pregnancy, studying what CB receptor is involved and to what degree these effects depend on prostaglandins and NO.

## Methods

### Data Availability

The data that support the findings of this study are available from the corresponding author upon reasonable request.

### Patient Inclusion

Patients within this study were included at 2 different locations: the Erasmus University Medical Center in Rotterdam, The Netherlands (Rotterdam cohort), for quantification of mRNA levels, protein levels, and vascular functional studies (outlined in the flowchart of Figure S1), and the University of Melbourne, Melbourne, Australia (Melbourne cohort), for quantification of mRNA levels.

#### Rotterdam Cohort

Patients in the Rotterdam cohort were included at the Erasmus University Medical Center in Rotterdam, the Netherlands, between January 19, 2023, and March 5, 2025. We included patients with uncomplicated term pregnancies undergoing elective cesarean section who did not use cannabis during gestation (referred to as control), healthy pregnant women who frequently (at least once a day, including throughout pregnancy, and continued until at least 4 weeks before delivery) used cannabis during gestation, and women with preeclampsia. In the healthy group, only women with a scheduled cesarean section were included. They provided written informed consent before delivery. Of each patient, the following characteristics were obtained: maternal age at delivery, maternal body mass index, GA at delivery, ethnicity, mode of conception, mode of delivery, parity, highest maternal mean arterial pressure during gestation, birth weight of the baby, fetal sex, and placental weight. The healthy group excluded patients with hypertensive disorders, fetal disorders or fetal abnormalities, FGR, multiple gestation, diabetes, gestational diabetes, placental abnormalities, cannabis use during gestation, viral or bacterial infections, or delivery before 37 weeks. The preeclamptic group included eoPE only, diagnosed according to the International Society for the Study of Hypertension in Pregnancy 2018 criteria, that is, new-onset hypertension after 20 weeks of gestation (systolic blood pressure ≥140 mm Hg or diastolic blood pressure ≥90 mm Hg) accompanied by proteinuria or other signs of maternal organ dysfunction or utero-placental dysfunction.^32^ The institutional medical ethical review board waived the need for specific approval for this study according to the Dutch law (MEC-2016-418 and MEC-2017-418), and all patients gave written consent for the use of their placenta and data before the experiments. (MEC‐2016‐418 and MEC‐2017‐418).

#### Melbourne Cohort

Patients in the Melbourne cohort were included at the Mercy Hospital, Melbourne, Australia, between June 14, 2012, and January 22, 2020. Here, we included preterm controls, women with FGR, women with eoPE and no FGR, and women with both eoPE and FGR. Diagnosis of eoPE was conducted in accordance with the American College of Obstetricians and Gynecologists guidelines.^33^ FGR was defined as having a birth weight below the 10th centile, which was defined as per Australian national birthweight percentiles by sex and GA.^34^ Preterm controls were obtained from women who delivered preterm (<34 weeks in this cohort) due to complications such as placenta previa or spontaneous preterm rupture of membranes. Patients were excluded from both case and control groups if they exhibited congenital abnormalities or congenital infection confirmed through histopathologic examination. Of each patient, the following characteristics were obtained from the electronic health records: maternal age at delivery, maternal body mass index, highest maternal protein/creatinine ratio, lowest maternal platelet count, the highest systolic and diastolic blood pressure during admission and at the booking visit, mean arterial pressure, parity, gravidity, GA, birth weight of the baby, fetal sex, and mode of delivery. For this cohort, ethics approval was granted by the Mercy Health Human Research Ethics Committee (R11/34), and participants presenting to the Mercy Hospital for Women (Melbourne, Australia) gave informed, written consent for sample collection.

### Tissue mRNA Levels

#### Rotterdam Cohort

Placentas were collected and processed within 30 minutes after delivery. The chorion, amnion, and decidua were removed, and central villous space biopsies were obtained from 3 different cotyledons without any sign of infarction or calcification and combined into 1 biopsy. Simultaneously, third-order branches of chorionic plate arteries were dissected and cleaned from surrounding tissue. All biopsies and vessel segments were snap-frozen in liquid nitrogen and stored at −80 °C until further analysis. For the quantification of mRNA, 30 mg of tissue was homogenized in cold RNA lysis tissue (Qiagen, Venlo, the Netherlands) with 2% dithiothreitol using a tissue homogenizer (Rotary homogenizer Bio-Gen PRO200; PRO Scientific Inc, Oxford, CT). RNA was extracted using the ISOLATE II RNA Mini kit (Bioline, London, United Kingdom) according to the manufacturer’s instructions and dissolved in RNase-free water. RNA was quantified with a NanoDrop ND1000 spectrophotometer (Thermo Fischer Scientific, Cleaveland, OH) and stored at −20 °C. cDNA was synthesized with a SensiFast cDNA synthesis kit (Bioline) from 400 ng of RNA according to the manufacturer’s instructions, diluted 1:10 in RNAse-free water, and stored overnight at 4 °C. Real-time quantitative polymerase chain reaction was performed with the SYBR green polymerase chain reaction kit (Bioline) on a CFX-96 light cycler (Bio-Rad, Hercules, CA) under the following conditions: initial denaturation at 95 °C for 8 minutes and 30 seconds, followed by 40 cycles comprising 15 seconds at 95 °C, and 60 seconds at 60 °C. Directly thereafter, a melt curve (10 seconds at 95 °C and then 5 seconds each at 0.5 °C increments between 65 °C and 95 °C) was run for each gene to confirm amplification of a single polymerase chain reaction product. The following genes were measured: *CB1*, *CB2*, *FAAH*, *DAGLα/β*, *MAGL*, *NAPE-PLD*, pre-mRNA processing factor 38A, peptidylprolyl isomerase A, and β-actin. Primer details are shown in Table S1. For all tested genes, the relative mRNA levels were calculated using the 2^−ΔΔCt^ method, normalized to the geometric mean of the mRNA level of 3 housekeeping genes: pre-mRNA processing factor 38A, peptidylprolyl isomerase A, and β-actin in the corresponding sample. Relative mRNA levels are presented as fold change.

#### Melbourne Cohort

In Melbourne, placental tissue samples were also collected and processed within 30 minutes after delivery.^35^ Samples were snap-frozen and stored at −80 °C for subsequent RNA extraction. Extraction of mRNA occurred in an identical manner as that in the Rotterdam cohort. cDNA was made using the Applied Biosystems high-capacity cDNA reverse transcriptase kit (Life Technologies, Carlsbad, CA) according to the manufacturer’s instructions using 400 ng of mRNA. The conversion was performed under the following conditions: 25 °C for 10 minutes, 37 °C for 60 minutes, and 85 °C for 5 minutes. cDNA was stored at −20 °C. A CFX384 (Bio-Rad) machine was used to perform quantitative polymerase chain reaction on the following protocol: 95 °C for 20 seconds, 95 °C for 1 second, and 60 °C for 20 seconds×40 cycles with a melt curve (65 °C for 5 seconds, 65 °C to 95 °C at 0.5 °C increments per 5 seconds) using the SYBR master mix Fast SYBR Master Mix (Thermo Fisher Scientific, Scoresby, Australia). The same (housekeeping) genes were tested, the same primer sets were used, and the same method was used for calculating the relative mRNA levels, which were equal to those in the Rotterdam cohort.

### Western Blotting

Western blotting was performed as described before^36^ in biopsies from control placentas to confirm the presence of CB1 and CB2 at the protein level. For each sample, 60 µg of protein was used. The following primary antibodies were used: anti-CB1 antibody produced in goat 1:500 (EB06945; Sanbio B.V., Uden, the Netherlands), anti-CB2 antibody produced in goat 1:500 (EB06946; Sanbio B.V.), and anti-β-actin 1:1000 (ab8229; Abcam, Cambridge, United Kingdom). To detect the protein signal, the membranes were incubated with a fluorescently labeled secondary antibody (ab97110; Abcam). Protein bands were visualized by the Odyssey Infrared Imaging System and analyzed in Image Studio Lite (LI-COR Biosciences).

### Wire Myography

Within 30 minutes after delivery of the placenta, third-order branches of chorionic plate arteries were dissected, cleaned from surrounding tissue, and stored in Krebs-Henseleit buffer (in mmol/L: NaCl 118, KCl 4.7, CaCl_2_ 2.5, MgSO_4_ 1.2, KH_2_PO_4_ 1.2, NaHCO_3_ 25, and glucose 8.3), aerated with 95% O_2_ and 5% CO_2_ at 4 °C. Within 24 hours after dissection, the vessels were cut into segments of 2 mm length and mounted in 6-mL organ baths (Danish Myograph Technology, Aarhus, Denmark). The baths contained Krebs-Henseleit buffer at 37 °C and were aerated with 95% O_2_ and 5% CO_2_. The tension of the segments was recorded in LabChart acquisition software (AD Instruments, Sydney, Australia). Each segment was normalized to 5.1 kPa to approximate in vivo conditions,^37–39^ and the internal diameter after normalization was recorded (Table S2). For the sake of clarity, we refer to the generation of tension as constriction and to relaxation as dilation. After obtaining a stable baseline, the maximum contractile response to 100 mmol/L KCl was determined in each segment. After KCl washout, segments were preconstricted with a concentration (range between 10 and 50 nmol/L) of the thromboxane A2 receptor agonist U46619 (Sigma-Aldrich, St. Louis, MO) that yielded ≈75% of the maximum KCl response. Then, concentration-response curves (0.1 nmol/L–30 µmol/L) to the endocannabinoids anandamide and 2-AG (both from Sigma-Aldrich), the selective CB1 receptor agonist HU-210 (Cayman Chemical, Ann Arbor, MI), or the selective CB2 receptor agonist HU-308 (Merck Life Science NV, Darmstadt, Germany) were constructed, without or with prior incubation for 30 min with the CB1 receptor antagonist AM251, the CB2 receptor antagonist AM630, the nonselective COX inhibitor indomethacin, the selective COX2 (cyclooxygenase 2) inhibitor celecoxib, or the NO synthase inhibitor NG-Nitro-L-Arginine Methyl Ester (L-NAME) (all from Sigma-Aldrich and all at 1 µmol/L).^8^ At the end of each concentration-response curve, the maximal response to the NO donor sodium nitroprusside (SNP, 100 µmol/L) was recorded. There were no statistical differences between vessel diameters and KCl responses in the various subgroups of all wire myography experiments (Table S2). None of the inhibitors affected vascular contractility during preincubation, and contractions induced by 50 nmol/L U46619 remained stable when exposing the vessel segments to the corresponding concentrations of the solvent of the eCBs (ethanol; Figure S2, n=4).

### Chemical Compounds

Anandamide and 2-AG were purchased as an ethanol solution (10 mmol/L). AM251 (1 mmol/L) was dissolved in ethanol. SNP (100 mmol/L) was dissolved in milliQ water. AM630 (1 mmol/L), HU-210 (10 mmol/L), HU-308 (10 mmol/L), indomethacin (1 mmol/L), celecoxib (1 mmol/L), and L-NAME (1 mmol/L) were dissolved in dimethylsulfoxide. All stock solutions were stored at −20 °C. Agonist dilution occurred in milliQ water.

### Statistical Analysis

Baseline characteristics of both the Rotterdam and Melbourne cohorts were analyzed and compared between groups using R (R Core Team, version 4.3.1, Vienna, Austria). The following packages were used: readxl, table1, ggplot2, ggpubr, ggtext, corrplot, and ggcorplot. The mRNA levels were compared between groups using a Kruskal-Wallis test with Dunn post hoc analysis (GraphPad Prism 8) using the term group as the control group for the Rotterdam cohort and the preterm group as the control group for the Melbourne cohort. A Pearson correlation analysis of the mRNA levels of ECS components and relevant clinical data of the Melbourne cohort was performed in R. For the wire myography experiments, relaxation was expressed as a percentage of the U46619 preconstriction. Sigmoid curve fitting (nonlinear fit) was applied to all concentration-response curves to estimate pEC_50_ values, that is, log_10_-transformed values at which the half-maximal response occurred (GraphPad Prism 8). The effects of the various blockers and the effect between control and eoPE or cannabis users were analyzed using a general linear model; repeated measures in IBM SPSS Statistics 25 (Chicago, IL); sphericity was assumed. Differences between SNP responses were analyzed using a nonparametric multiple comparison test (Kruskal-Wallis test) in GraphPad Prism 8. Data are presented as median (range) unless described otherwise, and a *P*<0.05 was considered statistically significant.

## Results

The analysis of the ECS mRNA data was done separately for the Rotterdam and Melbourne cohorts, of which descriptives are provided in Tables S3 and S4, respectively. The clinical data belonging to the placentas used in the wire myography experiments are shown in Table S5, while those belonging to the vascular CB1/CB2 mRNA levels studies are shown in Table S6. The use of cannabis (and other drugs, as well as their smoking behavior) in women frequently using cannabis is outlined in the Table. As expected, eoPE women in the Rotterdam cohort displayed a higher mean arterial pressure, a lower birthweight and placental weight, and a lower GA. Maternal characteristics of the 5 cannabis users were not different from the healthy control group. Two of these 5 women delivered vaginally, versus none in the other groups. In the Melbourne cohort, GA was identical in the 4 subgroups, and mean arterial pressure was elevated in eoPE women, with or without FGR.

**Table.**
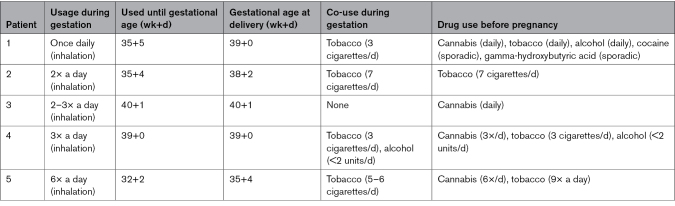
Drug and Alcohol Use, as well as Smoking Behavior by the 5 Women Who Used Cannabis During Their Pregnancy

### Placental mRNA Levels of Endocannabinoid Components Are Altered With Preeclampsia and Cannabis Use

#### Rotterdam Cohort

**Figure 2.**
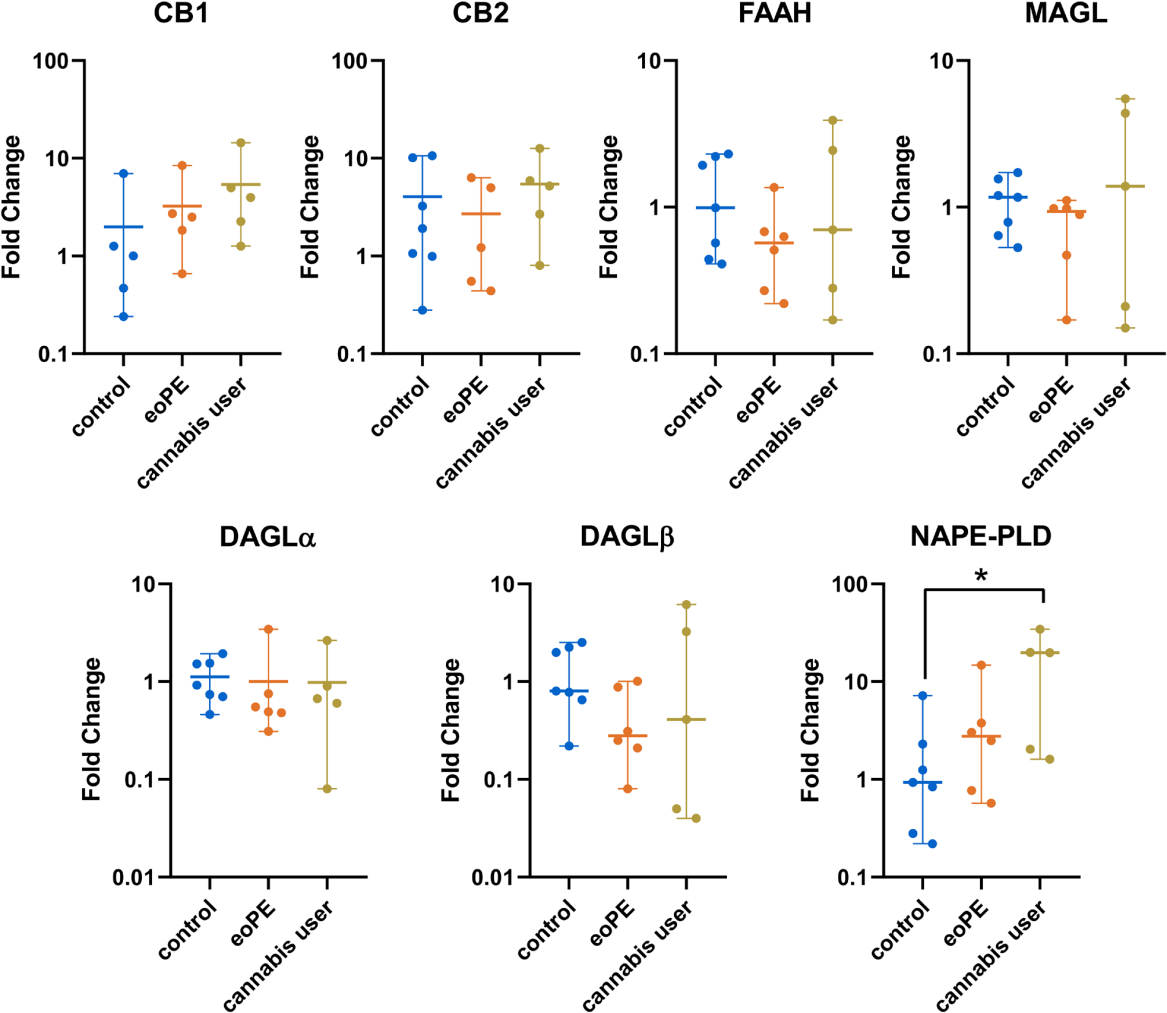
**mRNA levels of endocannabinoid system components in term control (n=7), early onset preeclampsia (eoPE, n=6), and cannabis user (n=5) central villous placental biopsies.** Data are shown as median and range and represent fold-change vs term control. *P<0.05 vs control (Dunn multiple comparison test). CB1 indicates cannabinoid receptor 1; CB2, cannabinoid receptor 2; DAGLα, 1,2-diacylglycerol lipase α; DAGLβ, 1,2-diacylglycerol lipase β; eoPE, early onset preeclampsia; FAAH, fatty acid amide hydrolase; MAGL, monoacylglycerol lipase; and NAPE-PLD, N-acyl phosphatidylethanolamine-specific phospholipase D.

Figure 2 shows the relative mRNA levels of *CB1*, *CB2*, *FAAH*, *MAGL*, *DAGLα/β*, and *NAPE-PLD* in healthy and eoPE placentas, as well as in placentas of cannabis users. The only statistically significant difference concerned placental *NAPE-PLD* mRNA, which was elevated in cannabis users compared with term control (*P*<0.05). Figure S3A shows that *CB1* and *CB2* mRNA could be detected in chorionic plate arteries of healthy term controls (n=6). Moreover, when studying 6 of the 7 available healthy placentas, we were able to confirm the presence of *CB1* and *CB2* at the protein level (Figure S3B).

#### Melbourne Cohort

**Figure 3.**
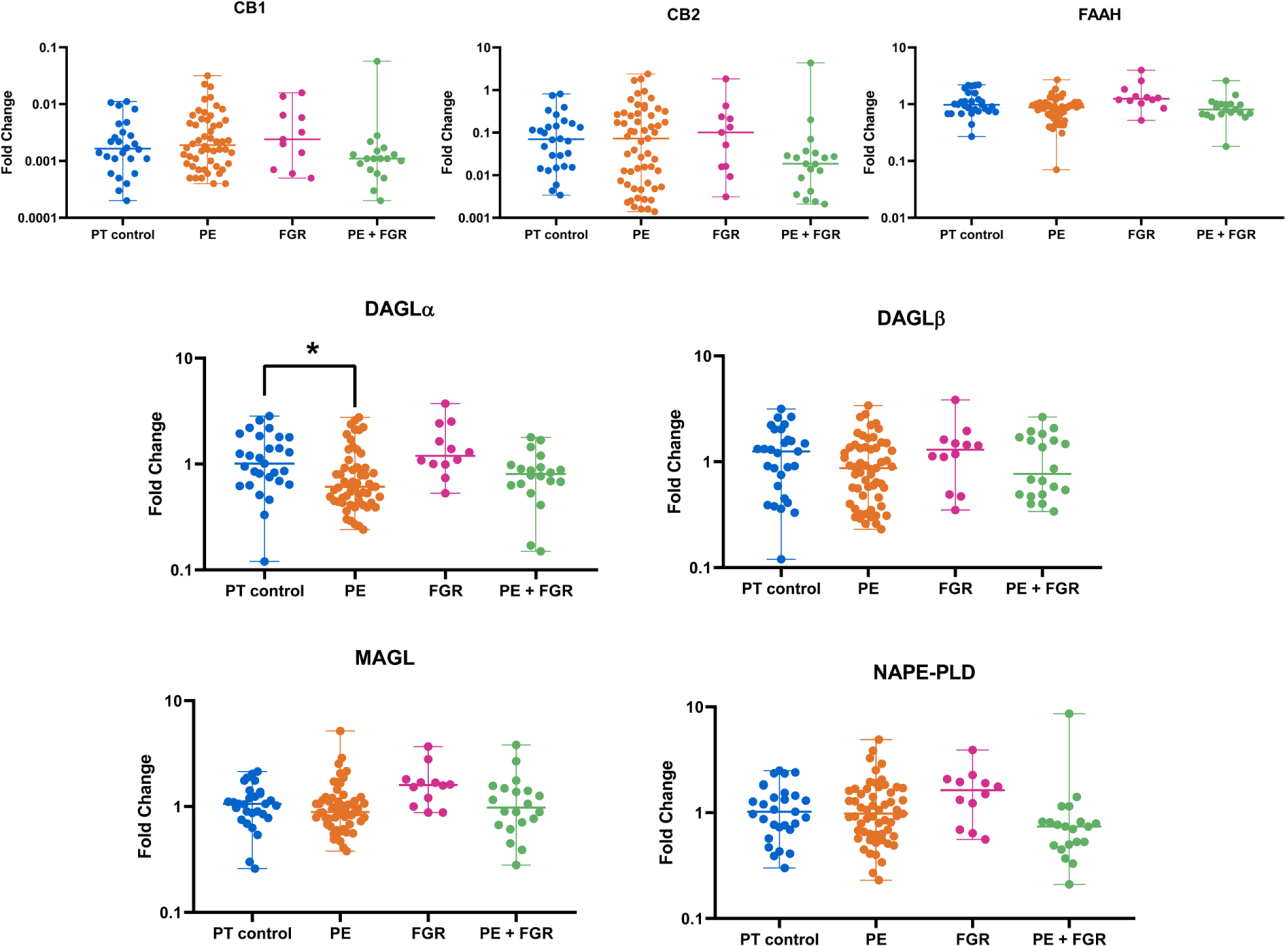
**mRNA levels of endocannabinoid system components in gestational age-matched control (PT; preterm, n=29), early onset preeclampsia (eoPE, n=57), fetal growth restriction (FGR, n=12), or eoPE+FGR (n=20) central villous placental biopsies.** Data (median and range) are shown as fold-change vs term control. *P<0.05 vs control (Dunn multiple comparison test). CB1 indicates cannabinoid receptor 1; CB2, cannabinoid receptor 2; DAGLα, 1,2-diacylglycerol lipase α; DAGLβ, 1,2-diacylglycerol lipase β; FAAH, fatty acid amide hydrolase; MAGL, monoacylglycerol lipase; and NAPE-PLD, N-acyl phosphatidylethanolamine-specific phospholipase D.

Figure 3 shows the relative mRNA levels of *CB1*, *CB2*, *FAAH*, *MAGL*, *DAGLα/β*, and *NAPE-PLD* in the 4 groups. *DAGLα* mRNA in eoPE placentas was lower versus preterm control (*P*<0.05). The mRNA levels of the CB1 receptor, the CB2 receptor, and NAPE-PLD were positively correlated, and this was also true for the mRNA levels of FAAH, DAGLα, DAGLβ, and MAGL. DAGLα and FAAH mRNA levels correlated negatively with blood pressure (Figure S4).

### Anandamide and 2-AG Relax Healthy Term and eoPE but Not Cannabis User Chorionic Plate Arteries

**Figure 4.**
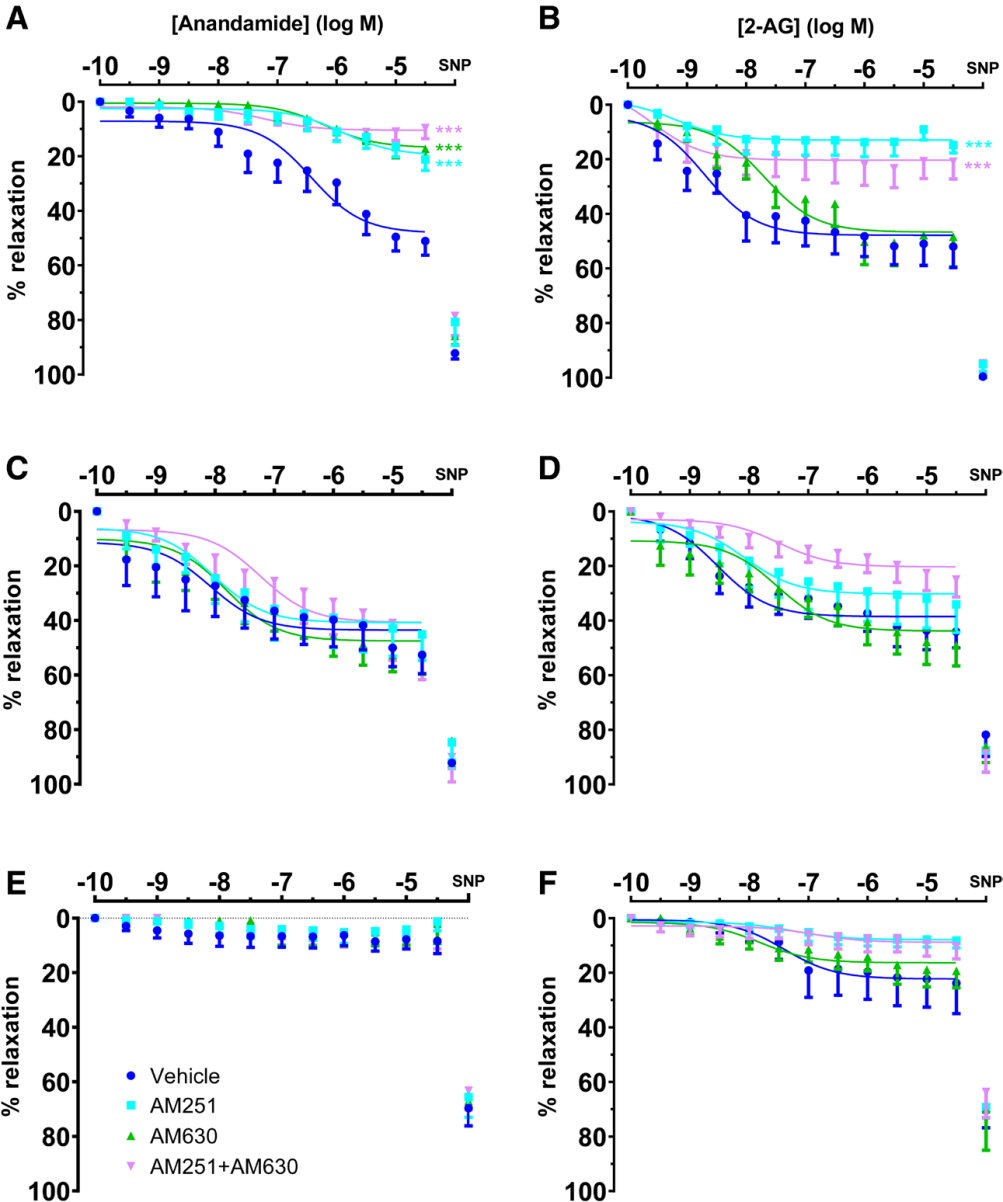
**Relaxations of preconstricted chorionic plate arteries to endogenous cannabinoid receptor agonists.Arteries obtained from healthy term (A and B, n=9), early onset preeclampsia (C and D, n=5–6) and cannabis user (E and F, n=5) placentas were exposed to anandamide and 2-arachidonoylglycerol (2-AG) in the absence (vehicle) or presence of the cannabinoid receptor type 1 receptor antagonist AM251, the cannabinoid receptor type 2 receptor antagonist AM630, or AM251+AM630.** The graphs also show the maximal relaxant response to 100 µmol/L sodium nitroprusside (SNP) obtained after finalization of the concentration-response curves. ***P<0.001 vs vehicle (general linear model; repeated measures).

Contractions were stable during vehicle (no eCB) application (n=4, Figure S2). In healthy term chorionic plate arteries, anandamide caused relaxation by a maximum of 51±5% (n=9; Figure 4A), with a pEC_50_ of 6.7±0.5. The CB1 receptor antagonist AM251 (n=9) and the CB2 receptor antagonist AM630 (n=9), when given separately, partially prevented the effect of anandamide (*P*<0.001 versus vehicle for both; Figure 4A), while in combination they virtually abolished the effects of anandamide (n=9; *P*<0.001 versus vehicle; Figure 4A). 2-AG relaxed healthy term arteries by maximum of 52±8% with a pEC_50_ of 9.2±0.9 (n=9; Figure 4B). AM251 (n=9; *P*<0.001 versus vehicle; Figure 4B), but not AM630 (n=9; Figure 4B), prevented this effect, while the effect of AM251+AM630 was identical to that of AM251 alone (Figure 4B). At the end of the concentration-response curves, SNP induced maximum relaxation in all healthy term placental vessels (95±14%; Figure 4A and 4B; Table S2).

Anandamide relaxed chorionic plate arteries from patients with eoPE to the same degree as healthy arteries (maximal relaxant effect 53±17%, pEC_50_ 8.9±1.2, n=6), but CB receptor antagonism with either AM251 alone (n=6), AM630 alone (n=6), or their combination (n=5) no longer affected this relaxation (Figure 4C). 2-AG also relaxed eoPE arteries to the same degree as healthy arteries (maximal relaxant effect 44±14%, pEC_50_ 7.47±0.5, n=5), and this relaxation too was no longer significantly affected by AM251, AM630, or their combination (Figure 4D). SNP induced the same maximum relaxation in the eoPE (88±18%) as in healthy term arteries (Figure 4C and 4D; Table S2).

Chorionic plate arteries from placentas of cannabis users no longer responded to anandamide, either with or without the CB receptor antagonists AM251 and AM630 (n=5; Figure 4E). These arteries only marginally responded to 2-AG (maximal relaxant effect 24±11%, pEC_50_ 7.1±0.4, n=5; Figure 4F). Yet, the effects of AM251, AM630, and their combination on 2-AG-induced dilation were similar in vessels of cannabis users versus healthy term vessels, although not statistically significant, most likely because of the modest effects of 2-AG. SNP relaxed the arteries of cannabis users to a lesser degree (*P*<0.0001) than healthy term arteries (68±22%; Figure 4E and 4F; Table S2).

### Selective CB1 and CB2 Receptor Agonists Do Not Relax Chorionic Plate Arteries From eoPE Placentas

**Figure 5.**
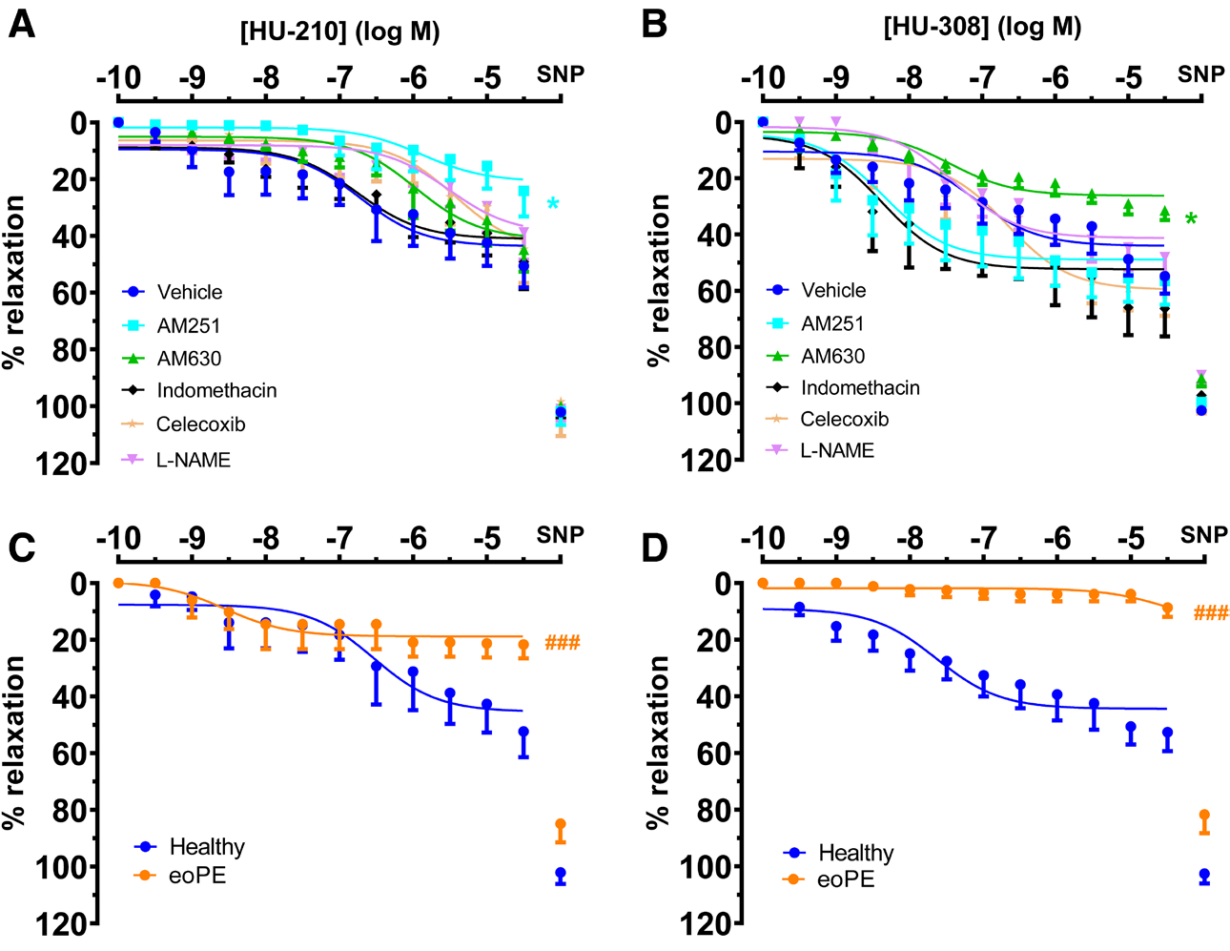
**Relaxations of preconstricted chorionic plate arteries to exogenous cannabinoid receptor agonists.** Arteries obtained from healthy term (**A** and **B**, n=6–8) and early onset preeclampsia (eoPE; **C** and **D**, n=3) placentas to the cannabinoid receptor type 1 agonist HU-210 or the cannabinoid receptor type 2 agonist HU-308 in the absence (vehicle) were exposed or presence of the cannabinoid receptor type 1 antagonist AM251, the cannabinoid receptor type 2 receptor antagonist AM630, the nonselective cyclooxygenase inhibitor indomethacin, the selective cyclooxygenase type 2 inhibitor celecoxib, or the nitric oxide synthase inhibitor NG-Nitro-L-Arginine Methyl Ester (L-NAME). The graphs also show the maximal relaxant response to 100 µmol/L sodium nitroprusside (SNP) obtained after finalization of the concentration-response curves. For the sake of clarity, **C** and **D** also contain the curves obtained in the presence of vehicle in healthy term arteries shown in **A** and **B**. *P<0.05 vs vehicle (general linear model; repeated measures), ###*P*<0.001 vs healthy (general linear model; repeated measures).

Since both anandamide and 2-AG also exert non-CB receptor-mediated effects, we additionally performed vascular experiments with the CB1-selective agonist HU-210 and the CB2-selective agonist HU-308. HU-210 relaxed healthy term arteries by a maximum ofd 50±7% with a pEC_50_ of 7.5±0.9 (n=6; Figure 5A). As expected, this relaxation was blocked by the CB1 receptor antagonist AM251 (n=6, *P*<0.05; Figure 5A) but not the CB2 receptor antagonist AM630 (n=6; Figure 5A). It was also not affected by indomethacin, celecoxib or L-NAME (n=6; Figure 5A). HU-308 relaxed healthy arteries by 55±6% (n=8; Figure 5B), with a pEC_50_ of 7.1±0.6. This relaxation was blocked by AM630 (n=8, *P*<0.05; Figure 5B), but not AM251 (n=6; Figure 5B). It was also unaffected by indomethacin (n=7; Figure 5B), celecoxib (n=7; Figure 5B), or L-NAME (n=6; Figure 5B). SNP induced maximum relaxation under all conditions (Figure 5A and 5B; Table S2). In arteries from eoPE women, neither HU-210 nor HU-308 induced dilation (Figure 5C and 5D; n=3), although SNP still relaxed these vessels to the same degree as those of the healthy term group.

## Discussion

This study focused on the placental ECS and CB-receptor-mediated vascular effects in healthy pregnant women, women with eoPE, and pregnant women who regularly used cannabis during their pregnancy. Cannabis use is on the rise during pregnancy, despite its potential association with FGR and preeclampsia and thus knowledge on the placental ECS in humans is warranted. Our GA-matched data indicate a downregulation of DAGLα in eoPE placentas, and an upregulation of NAPE-PLD in cannabis users. As illustrated in Figure 1, the former will diminish the generation of 2-AG, while the latter will upregulate the generation of anandamide. Given the relaxant effects of these agonists on chorionic plate vessels, alterations in their concentrations are likely to affect vasoreactivity. Indeed, we observed that in eoPE vessels, the effects of these agonists were no longer mediated by CB receptors, while in cannabis users, their effects were either greatly diminished (2-AG) or entirely absent (anandamide). Moreover, relaxation induced by the endothelium-independent NO donor SNP was also diminished in cannabis users but not in eoPE. Taken together, these data indicate that a disturbed ECS might be a player in eoPE, potentially by indirectly enhancing constriction. Moreover, cannabis use directly disturbs the placental ECS, thus explaining its association with FGR and preeclampsia.

It has already been reported that the ECS contributes to embryo implantation and trophoblast differentiation.^18,19^ Our mRNA quantification data now support that the human placenta is capable of generating all components of the ECS independently, implying that the generation of anandamide and 2-AG may occur at placental tissue sites and that these agonists can then bind to placental CB receptors. At least 1 location of these receptors, given our observations, appears to be the placental vasculature. Furthermore, the mRNA levels of multiple ECS components were highly correlated, suggesting identical upstream regulatory pathways. These data do not exclude an additional role for maternal anandamide and 2-AG, which might be taken up from circulating blood.

Placental DAGLα downregulation was significant only in women with eoPE without FGR versus GA-matched preterm controls. A similar tendency was observed in women with eoPE+FGR but not in women with FGR only. When making a comparison versus healthy term pregnancies, again DAGLα mRNA levels tended to be lower in eoPE women, although significance was not reached. Future studies should address whether DAGLα downregulation truly lowers placental 2-AG levels.

Given the dilator effects of both 2-AG and anandamide, lowering their concentration might result in vasoconstriction and diminished placental blood flow. Remarkably, our data indicate that anandamide- and 2-AG-mediated relaxant effects in chorionic plate vessels obtained from eoPE women no longer involved CB1 or CB2 receptors, while in healthy pregnant women their effects relied entirely on CB1 plus CB2 receptors (anandamide) or CB1 receptors exclusively (2-AG). In support of the absence of CB receptor-mediated effects in eoPE, both the selective CB1 receptor agonist HU-210 and the selective CB2 receptor agonist HU-308 also no longer induced any effect in chorionic plate arteries from women with eoPE, while they did dilate vessels from healthy term women, reaching maximal effects that were comparable to those of anandamide and 2-AG. The relaxant effects of these selective agonists did not rely on NO or prostaglandins. Importantly, in agreement with earlier observations,^40^ the relaxant effects of the NO donor SNP were fully intact in chorionic plate arteries of eoPE women. This indicates that the ECS disturbance in this condition specifically concerns the CB receptors and not relaxation in general. One proposal to explain these findings is CB1 receptor desensitization due to excessive anandamide uptake from maternal blood.^41^ Given that the relaxant effects of anandamide and 2-AG remained intact in eoPE but no longer involved CB receptors, they must have been mediated via alternative mechanisms. This may include the TRPV1 channel for anandamide^4,42^ or GPR55 for 2-AG.^7^ GPR55 regulates α1A-adrenergic receptor-mediated vasoconstriction.^43^ Another potential player linked to the ECS is PPARγ, which modulates cGMP.^44,45^

In apparent contrast with our data, Abán et al^46^ observed NAPE-PLD upregulation in women with preeclampsia. However, these authors did not make a distinction between eoPE and late-onset preeclampsia, and this could explain the discrepancy. We did see NAPE-PLD upregulation in cannabis users. This might be considered beneficial, as it would increase anandamide, thereby reducing maternal stress.^47^ Yet, high levels of anandamide also reduce the growth of sheep embryos,^48^ and this could underlie the relationship between cannabis use and FGR.^49^ According to this concept, anandamide induces apoptosis and inhibits cell proliferation via CB1 receptors. Our data now extend this observation by showing that cannabis use in humans resulted in the total absence of endogenous ECS responses in chorionic plate vessels. Combined with diminished NO responses, this is suggestive of vascular dysfunction. Similarly, cannabis use in rats has been reported to result in endothelial dysfunction,^50^ while cannabis use by pregnant women is associated with preeclampsia, which is also characterized by endothelial dysfunction.^51^ Our data, however, rather point to a defect at the level of the vascular smooth muscle cells, since this is where exogenous NO acts. Clearly, the CB receptor-independent pathways that allowed endogenous ECS agonists to still induce relaxation in eoPE are no longer functional following exposure to exogenous ECS stimulators. Most likely, as this concerned cannabis inhalation of up to 3× per day, a higher degree of ECS stimulation was reached as compared with the situation in eoPE, potentially also downregulating these alternative pathways. To fully appreciate the implications of cannabis use during pregnancy, studies on the placental passage of the exogenous cannabis components (cannabidiol and Δ^9^-tetrahydrocannabinol) are required, combined with knowledge on the levels of endogenous CB receptor agonists in placental tissue after cannabis use. A caveat is that the cannabis-using women in our study also smoked cigarettes, drank alcohol, and occasionally used cocaine and gamma-hydroxybutyric acid. Thus, a role for these factors cannot be excluded at present. Moreover, 2 of the cannabis-using women delivered vaginally (versus none in the other groups), and it has been reported that vaginal delivery might affect the circulating eCB levels.^52^ However, since the vascular reactivity data in the cannabis users were highly consistent, it seems unlikely that this has played a role.

## Perspectives

Our data indicate a disturbance of the ECS in eoPE, implying a role for this system in preeclampsia. Furthermore, cannabis use during pregnancy resulted in the complete disappearance of the vasodilator effects of endogenous ECS components in placental vessels, combined with diminished NO signaling in vascular smooth muscle cells. These data warn against the use of cannabis and cannabis-containing oils in pregnancy. They could also explain why such use is associated with FGR and preeclampsia. Here it is important to note that paracetamol is metabolized to AM404,^53^ which may act as a CB receptor agonist and additionally inhibits the degradation of anandamide.^53^ Hence, it could be considered a safe alternative. Although the n-numbers in this study were small, the vasoreactivity data were highly consistent. Yet, cannabis was often not the only drug used by these women. Future studies should investigate to what degree maternal use of cannabidiol and Δ^9^-tetrahydrocannabinol results in their accumulation in the fetus and how they affect placental development. Such research should also gain more insight into the dose, mode, and timing of such intake, as this might moderate their effects. It is crucial that women are asked about cannabis use during preconception and pregnancy, are counseled on the risks of continued use in pregnancy, and are referred to addiction care if needed.

## Article Information

### Sources of Funding

M.S. Harhangi has been supported by a grant of the Sophia Foundation (WAR23-28).

### Disclosures

None.

### Supplemental Material

Tables S1–S6

Figures S1–S4
